# Microtube Array Membrane (MTAM)-Based Encapsulated Cell Therapy for Cancer Treatment

**DOI:** 10.3390/membranes10050080

**Published:** 2020-04-26

**Authors:** Chee Ho Chew, Chih-Wei Lee, Wan-Ting Huang, Li-Wei Cheng, Amanda Chen, Tsai-Mu Cheng, Yen-Lin Liu, Chien-Chung Chen

**Affiliations:** 1 Graduate Institute of Biomedical Materials & Tissue Engineering, Taipei Medical University, Xinyi District, Taipei 11031, Taiwan; chchew88@gmail.com (C.H.C.); q147555251@gmail.com (C.-W.L.); sandyhuang@mtamtech.com (W.-T.H.); harry51513@gmail.com (L.-W.C.); 2 Department of Biochemistry, University of Washington, Seattle, WA 98195, USA; mandycookie13@gmail.com; 3 The PhD Program for Translational Medicine, Taipei Medical University, Taipei 11052, Taiwan; tmcheng@tmu.edu.tw; 4 Department of Pediatrics, Taipei Medical University Hospital, Taipei 11052, Taiwan; ylliu@tmu.edu.tw; 5 International Ph.D. Program in Biomedical Engineering, College of Biomedical Engineering, Taipei Medical University, Taipei 110, Taiwan; 6 International PhD Program for Cell Therapy and Regeneration Medicine, College of Medicine, Taipei Medical University, Taipei 110, Taiwan; 7 Ph.D Program in Biotechnology Research and Development, College of Pharmacy, Taipei Medical University, Taipei 110, Taiwan

**Keywords:** encapsulated cell therapy (ECT), hybridoma, cancer, microtube array membrane (MTAM), electrospinning

## Abstract

The treatment of cancer has evolved significantly in recent years with a strong focus on immunotherapy. Encapsulated Cell Therapy (ECT) for immunotherapy-based anti-cancer treatment is a unique niche within this landscape, where molecules such as signaling factors and antibodies produced from cells are encapsulated within a vehicle, with a host amount of benefits in terms of treatment efficacy and reduced side effects. However, traditional ECTs generally lie in two extremes; either a macro scale vehicle is utilized, resulting in a retrievable system but with limited diffusion and surface area, or a micro scale vehicle is utilized, resulting in a system that has excellent diffusion and surface area but is unretrievable in the event of side effects occurring, which greatly compromises the biosafety of patients. In this study we adapted our patented and novel electrospun Polysulfone (PSF) Microtube Array Membranes (MTAMs) as a ‘middle’ approach to the above dilemma, which possess excellent diffusion and surface area while being retrievable. Hybridoma cells were encapsulated within the PSF MTAMs, where they produced CEACAM6 antibodies to be used in the suppression of cancer cell line A549, MDA-MB-468 and PC 3 (control). *In vitro* and *in vivo* studies revealed excellent cell viability of hybridoma cells with continuous secretion of CEACAM6 antibodies which suppressed the MDA-MB-468 throughout the entire 21 days of experiment. Such outcome suggested that the PSF MTAMs were not only an excellent three-dimensional (3D) cell culture substrate but potentially also an excellent vehicle for the application in ECT systems. Future research needs to include a long term *in vivo* >6 months study before it can be used in clinical applications.

## 1. Introduction

In the modern era, diseases continue to plague mankind despite the advances in medical technology. Among the myriad of diseases, cancer remains one of the leading cause of death of patients [1]. In the past few decades, various research works have significantly enhanced our understanding of the mechanism and the regulation of the patient’s immune system against cancer [2]. Despite the advances, challenges such as the inability to accurately predict treatment response, the need for accurate and clinically significant biomarkers, extremely high treatment cost of immunotherapy and the knowledge on matters pertaining to the development of resistance to immunotherapy in cancer treatment continues to hamper the delivery of effective immunotherapy anti-cancer treatment to patients [3,4,5]. In order to address these challenges, multiple efforts have been made to develop innovations in the area of strategies to prevent, reduce and ultimately eliminate cancer recurrences/incidences, personalize biomarkers that can more accurately predict or monitor treatment progress, create combination therapies that can significantly improve treatment efficacy and safety and reduce the financial burden associated with cancer treatment [3,6,7].

Another leading area of development is encapsulated cell therapy (ECT). ECT is the process where cells that naturally produce therapeutic molecules are encapsulated within a semipermeable membrane, which allows the inflow diffusion of nutrients and oxygen into the membrane and the outflow diffusion of waste and therapeutic compounds. More importantly, the membrane that encapsulates the cells also serves as a protection barrier against adjacent foreign cells and also against the host immune system [8]. Interestingly, in addition to improving cancer treatment outcome, ECT is capable of delivering therapeutic molecules for a prolonged period without the need of repeated treatments, which could be beneficial in terms of the reduction of the dosage required and frequency that can significantly benefit cancer patients receiving anti-cancer drugs, which are often toxic [8,9,10,11,12]. Additionally, ECT can be applied directly by implantation adjacent to the therapeutic target, and this would significantly minimize the systemic concentration of these therapeutic compounds, thereby potentially adverting any potential side effects [11,13,14]. This strategy have been widely adopted by several research groups in various cancers demonstrating a potential alternative to traditional systemic anticancer treatments that are often plagued with side effects [11,12,15,16]. 

As with any treatment, the biosafety of cancer patients is of utmost importance. ECT systems offers a potential solution as the membrane surrounding the encapsulated cells prevents out of control growth and migration; moreover, if by any chance problems occur throughout the course of treatment, the entire membrane can be removed, thereby effectively stopping the treatment [11,17]. This is especially critical with the current shift of focus from conventional cancer treatment to immunotherapy/cell-based treatments, which could bring about both the promise of a potential cancer cure and the possibility of adverse side effects. Current knowledge within these fields are by no means adequate. 

Currently, several research groups have conducted studies or clinical trials relating to the treatment of cancers using ECT systems. Lang et al. demonstrated that the encapsulation of hybridoma cells producing antibodies directed against the domain of the p15E domain that is responsible for tumor suppressive effects within an alginate capsules which revealed a prolonged survival in mouse leukemia models [18,19]. Similarly, prolonged survival were also observed in the case of encapsulated Inducible nitric oxide synthase (iNOS) expressing cells within alginate microcapsules in DLD-1 human colon adenocarcinoma xenograft models, while a curative outcome was obtained in the case of SKOV-3 ovarian carcinoma cell line [18,20]. In the case of glioblastomas, work by Read et al. demonstrated anti-angiogenesis protein (endostatin) released by genetically engineered cells that were encapsulated within sodium alginate disrupted the microenvironment of tumors through inducing hypoxia, apoptosis and anti-angiogenesis effects, which ultimately led to the prolonged survival in adult inbred BD-IX rats models [21]. In another ECT-related work on glioblastomas, endostatin producing HEK 293 cells that were encapsulated within sodium alginate resulted in reduced tumor growth, perfusion and invasion [13,18]. Work by these groups suggested that there is significant potential that is yet to be fully realized in the use of ECT in cancer therapy.

Despite the promising outcome outlined above, these ECT systems generally fall within two major categories, namely, being in the size of macroscale, which results in a small diffusion surface area, or being in the microscale, which results in a large surface area. Hence, the existing systems currently utilized in ECTs are a tradeoff of either being in the macroscale, which results in it being recoverable but having a long diffusion distance from the surface to cells housed within and a low effective surface area, or being in the micron scale, which results in it being non-recoverable but with very short diffusion distance (<50 µm) and excellent effective large surface for the diffusion of nutrients, gases and target therapeutic products that potentially result in excellent cellular growth and maintenance [22]. Therefore, the inability to retrieve the encapsulated cells should the treatment produce undesirable/side effects is a major stumbling block in current systems. The ability to retrieve ECT systems would prove essential in future clinical applications by increasing the biosafety of patients [17]. In view of these challenges, we strive to adopt our internally developed, new class of hollow fibers called Microtube Array Membranes (MTAMs). MTAMs consists of ultra-thin, one-to-one connected microtube fibers that are arranged in an arrayed parallel formation [23]. Compared to traditional hollow fibers (HFs), the lumen walls of MTAMs are 100 times thinner (2–3 µm) than that of traditional HFs. Furthermore, the ability to modify the microstructures of the MTAMs allowed us to apply our MTAMs in various applications ranging from tissue regeneration [24,25,26], green energy [27], fermentation [28], bioreactor etc. 

Among the more recent and noteworthy developments in the use of MTAMs is the application of it in anticancer drug screening for personalized medicine that was recently published [29]. In this study, primary tumor biopsies were derived from patients and encapsulated within polylactic acid (PLLA) MTAMs, subcutaneously implanted in Balb/C mice, after which the desired range of anti-cancer agents were administered accordingly. Within a very short and clinically practical duration of 14 d, a particular cancer agent most effective against the patient’s cancer can be identified and this information pass on to oncologist for treatment. 

In contrast, the current study is focused on the encapsulation of hybidomas within PSF MTAMs as a potential ‘middle path’ ECT solution that brings tremendous value to potential future patients by incorporating the ability to be recoverable in an event of side effects, while providing a very short diffusion distance of no more than 30 µm from the surface of the MTAMs, which is well within the 50 µm threshold of nutrient and gases diffusion, as well as having high homogenously nanoporous pores along the entire surface. The combination of the above outline factors when combined with its excellent biocompatibility and excellent trans lumen wall diffusion makes it a potentially interesting platform to be explored in ECT systems [28].

## 2. Materials and Methods 

### 2.1. Preparation of Electrospun Polysulfone (PSF) MTAMs

PSF beads (Sigma, St. Louis, MO, USA), together with polyethylene glycol (Sigma-Aldrich, St. Louis, MO,, USA), were dissolved in a co-solvent of N,N-dimethyl formamide (DMF; Tedia, OH, USA) and dichloromethane (DCM; Mallinckrodt, St. Louis, MO, USA) at a ratio of 7:3 until homogenous. The resulting polymer solution was electrospun as the ‘shell solution’ together with a ‘core solution,’ which consisted of a mixture of polyethylene glycol (Sigma-Aldrich, USA) and polyethylene oxide (Sigma-Aldrich, St. Louis, MO, USA), at a voltage of between 5–7 kV under ambient conditions. The resulting PSF MTAMs were then retrieved and washed in double distilled water (ddH_2_O) and air dried. Next, the PSF MTAMs were then examined with a scanning electron microscope (SEM; Hitachi, Tokyo, Japan) and the microstructure parameters quantified.

### 2.2. PSF MTAMs as a Culture Substrate for Hybridoma Cells 

Hybridoma cells were provided by Professor Cheng Tsai-Mu of the Ph.D. Program for Translational Medicine, Taipei Medical University. Hybridoma cells in 50 mL conical tubes were centrifuged 1200 RPM for 5 min. The supernatant was discarded and the resulting pellets were retrieved and suspended in DMEM medium to achieve a cell density of 2 × 10^5^ cells/10 µL that was optimized in previous work [29]. Next, the respective PSF MTAMs that were pre-sterilized with ultraviolet (UV) light and cut into the size of 0.5 cm × 2.0 cm were used to siphon 10 µL of cell suspension. The respective ends of the PSF MTAMs were folded over and crushed with a tweezer, forming a tight seal (Supplementary Materials Figure S1). The cell loaded PSF MTAMs were then transferred into a six-well cell culture dish, which contained 2 mL of DMEM medium supplemented with fetal bovine serum (FBS) each, and incubated in 5% CO_2_ atmosphere. At predetermined time points, the cell viability was determined via MTT assay. Briefly, the cell containing PSF MTAMs were retrieved and incubated in 0.5 mg/mL MTT solution (Sigma, St. Louis, MO, USA) at 37 degrees Celsius for 60 min. Next, the substrate was washed with 100 µL of dimethyl sulfoxide (DMSO; Sigma, St. Louis, MO, USA) and the absorbance was determined with an ELISA reader (TECAN, Mannerdorf, Switzerland; 570 nm).

### 2.3. Production and Quantification of CEACAM6 Antibody

The culture of hybridoma cells were generally similar to those described above. Cultured cells suspensions were retrieved at pre-determined time points and centrifuged 1200 RPM for 5 min. The supernatant was collected and stored at 4 degrees Celsius for subsequent CEACAM6 antibody quantification studies. The quantification process was conducted in accordance to the procedure outlined by Cloud Clone Corp (Katy, Texas, USA). Briefly, 50 µL of cell suspension samples were transferred into the respective wells in a 96 well plate. Next, detection reagent A was added and incubated at 37 degrees Celsius for 60 min, and after which the samples were washed three times with ddH_2_O. The detection reagent B was then added into the respective wells and the incubation step was repeated. Then, it was washed five times with ddH_2_O. Next, 90 µL of the substrate solution was added to the respective wells incubated at 37 degrees Celsius for 20 min. After the incubation period, 50 µL of stop solution was added and the absorbance values were determined with an ELISA reader (TECAN, Mannerdorf, Switzerland; 450 nm).

### 2.4. In Vitro Culture of A549, PC3 and MDA-MB-468 Cancer Cell Lines in Medium Conditioned with CEACAM6 Antibody

A549 (provided by Professor Tseng Ching-Li of the College of Biomedical Engineering, Taipei Medical University), PC3 (Professor Lin Chun-Mao of the TMU Research Center of Cancer Translational Medicine) and MDA-MB-468 (provided by Professor Cheng Tsai-Mu of the Ph.D. Program for Translational Medicine, Taipei Medical University) (2 × 10^5^ cells/10 µL) were prepared and seeded into respective wells in six-well culture plates containing DMEM medium with 10% FBS and conditioned with 2 mL of supernatant derived from the centrifuged culture of hybridoma cells 24 h post seeding. The respective cancer cell lines were incubated at 37 degrees Celsius at 5% CO_2_ atmosphere. At predetermined time points, the cell viability of the respective cancer cell lines was determined using MTT assay at a wavelength of 570 nm.

### 2.5. In Vivo Studies of the Effects of CEACAM6 Antibody Against PC3 and MDA-MB-468 Cancer Cell Lines in Balb/C Mouse Model

All animal models conducted in this study were approved by an animal research committee (LAC-2016-0450) and conducted in accordance to the guidelines outlined by the Association for Assessment and Accreditation of Laboratory Animal Care including facility (protocol number LAC-2U14-U193). Balb/C mouse (30 mice, 6–7 weeks old) that were free from pathogens at the time of use were sourced from BioLASCO Taiwan Co., Ltd. and housed in TMU Laboratory Animal Center (Taipei, Taiwan). The respective cancer cell lines and hybridoma cells were cultured as outlined above and siphoned into the respective PSF MTAMs at a cell density 2 × 10^5^ cells/10 μL, and the ends were sealed. Next, the cell loaded PSF MTAMs were cultured for 24 h in DMEM medium at 37 degrees Celsius in 5% CO_2_ atmosphere. Anesthetized (methoxyflurane [Pitman-Moore, Inc., USA]) mice were prepared by shaving their backs, and 2 cm incisions were made. Next, the PSF MTAMs loaded with the respective cells were placed on a laboratory spoon and inserted into the skin incision, which was closed with a skin staple. This process was repeated three times on the backs for each mouse for hybridoma cells, PC 3 and MDA-MB-468 cancer cell lines. At predetermined time points (day 7, 14 and 21), the respective cell containing PSF MTAMs were retrieved and the respective cell viabilities determined via MTT assay. The histopathology of the respective cells was also determined via Haemotoxylin and Eosin (H&E) staining. The resulting images were analyzed (surface area) and quantified by Image J (NIH, USA). 

## 3. Results

**Figure 1:**
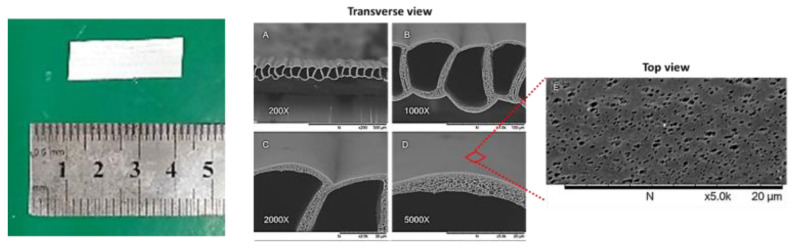
Macroscopic view of the electrospun polysulfone (PSF) Microtube Array Membranes (MTAMs) utilized in this study (left). SEM images of PSF MTAMs. The unique microstructures of the PSF MTAMs, which consisted of one-to-one connected, ultrathin, submicron scale hollow fibers, can clearly be observed from the transverse view (**A**–**D**). The individual lumen dimensions registered a value of 60.458 ± 0.488 μm × 39.549 ± 0.616 μm (Height × width; B), while the lumen wall thickness registered a value of 3.0 ± 1.0 μm (D). Top view of the PSF MTAMs revealed homogenously distributed pores along the entire surface of the PSF MTAMs (**E**), while the pore sizes were about 30 nm.

The PSF MTAMs were successfully electrospun at the above outlined parameters. SEM images (Figure 1) of the microstructure of the PSF MTAMs revealed one-to-one individually connected hollow fibers that were homogenously porous with the mean pore size of about 30 nm. The lumen walls registered a thickness of about 3.0 ± 1.0 μm. The combinations of these unique microstructures translated to a culture substrate, which was sufficiently large to house cells, encapsulate them within, allow the diffusion of molecules across the membrane and, more importantly, prevent the escape of the cells, while keeping the host immune system at bay. The individual dimensions were approximately 60.458 ± 0.488 μm × 39.549 ± 0.616 μm (height × width). On a macro scale, the semi translucent PSF MTAMs provided easy observation of the siphoning process of the cell suspension. 

**Figure 2:**
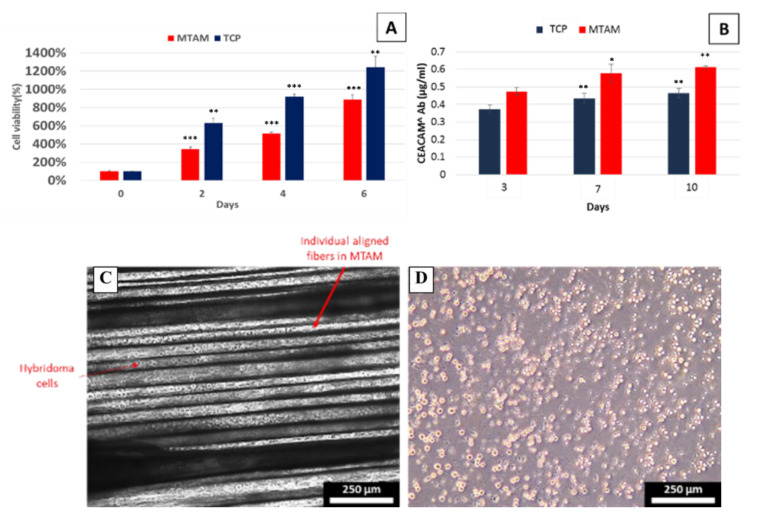
Cell viability of hybridoma cells cultured on TCPs vs. MTAMs (**A**) and CEACAM6 antibody levels produced by hybridoma cells when cultured on TCPs vs. MTAMs (**B**). Evidently, the higher levels of CEACAM6 antibody was registered by the hybridoma cells cultured within MTAMs as opposed to those cultured on standard TCPs. The antibody levels also do not directly correlate with the higher cell viability of hybridoma cells cultured on TCPs (A), and this was possibly substrate (MTAM) induced, since all other parameters were fixed. Optical image of hybridoma of day 6 encapsulated within MTAMs (**C**) within the respective lumens of MTAMs and hybridoma cultured on TCPs (**D**).

Additionally, it allowed for easy observation of the cells housed within the PSF MTAMs with a standard optical microscope (Figure 2). In the *in vitro* cultures (Figure 2A), the cell viability of hybridoma cells cultured within the PSF MTAMs registered a lower cell viability when compared to those cultured within the TCPs (Tissue Culture Plates). Despite the lower cell viability, the hybridomas that were cultured within the PSF MTAMs registered a significantly higher CEACAM6 antibody production and this suggested that the unique microstructures of the PSF MTAMs, which provided an excellent three-dimensional (3D) substrate and, when combined with the topographical features that were derived from the pores, indirectly affected the regulation and production of CEACAM6 antibodies. The trend was observed from the start of the *in vitro* culture of hybridoma cells within PSF MTAMs, and consistently increased throughout the entire culture duration of 10 d, which suggested that the PSF MTAMs were superior to that of TCPs when it comes to eliciting functional responses from cells cultured within.

**Figure 3:**
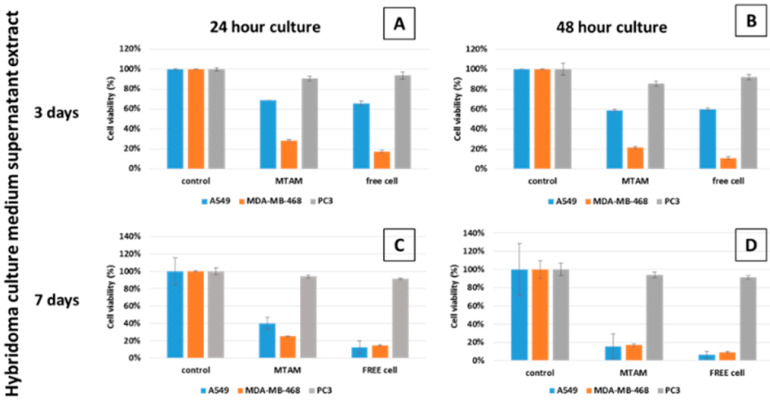
24 h (**A** and **C**) and 48 h (**B** and **D**) of the cell viability of the respective cancer cells lines A549, MDA-MB-468 and PC 3 when treated with hybridoma cell culture supernatant extracts. PC 3 cancer cell lines registered a minimal reduction of cell viability across all treatment, as it lacks the necessary binding sites for CEACAM6 antibody. In the case of A549 and MDA-MB-468, both cancer cell lines possess CEACAM6, which resulted in reduced cell viability when cultured in increased CEACAM6 antibody contained medium (supernatant extract from hybridoma culture medium after 3 days: 0.473 µg/mL; and 7 days: 0.576 µg/mL). CEACAM6 antibody levels were extracted from Figure 2B.

After culturing hybridoma cells in PSF MTAMs for 24 h and 48 h, the respective supernatant of these cell cultures were easily isolated from the pellet via centrifugation. The resulting supernatants were added to the culture mediums of the respective cancer cell lines under *in vitro* conditions. The negative control groups of all cancer cell lines, regardless of experimental group, revealed excellent viability (Figure 3). When comparing the cells cultured within the PSF MTAMs or TCPs, the A549, MDA-MB-468 and PC 3 cancer cell lines registered relatively similar viabilities across all treatment groups, which suggested that the PSF MTAMs did not hamper the diffusion of nutrient, waste or the antibody diffusion from the surrounding medium into the respective cells within the PSF MTAMs. PC 3 cancer cell line, which lacked the necessary CEACAM6 sites, was not susceptible to CEACAM6 antibodies; the results echo this, revealing a minimal reduction in terms of cell viability (Supplementary Materials Figure S3; [30]). Conversely, both A549 and MDA-MB-468 cancer cell lines were susceptible to the effects of CEACAM6 antibodies, with MDA-MB-468 possessing more binding site as compared to those found in A549 cancer cell line. The culture of A549 and MDA-MB-468 cancer cell lines in medium that contained supernatant of 48-hour hybridoma cell culture medium revealed a lower cell viability, and this value remained suppressed throughout the culture duration from day 3 to day 7.

**Figure 4:**
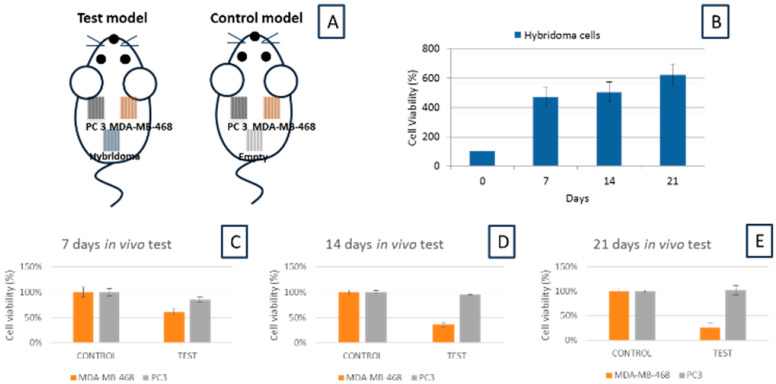
Schematic description of experimental groups in this *in vivo* study (**A**). Cell viability of hybridoma cells encapsulated within PSF MTAMs that were implanted subcutaneously for up to 21 days (**B**), with the cell viability continuously increased throughout the culture duration. Cell viability of the respective cancer cells lines PC3 and MDA-MB-468 for 7 d (**C**), 14 d (**D**) and 21 d (**E**) under *in vivo* culture conditions. Control groups demonstrated no reduction in cell viability, which demonstrated that the PSF MTAMs were a suitable cell culture substrate that did not hamper cell viability, while a significant reduction in cell viability of MDA-MB-468 cancer cell line was recorded. This reduction, when combined with the increased viability of hybridoma cells (B), suggested that the PSF MTAMs encapsulated hybridoma cells continuously released CEACAM6 antibodies, while maintaining excellent hybridoma viability throughout the entire culture duration.

This echoed the results in Figure 2B, which showed that the CEACAM6 antibody levels progressively increased with time and the extract from a later hybridoma cell culture will have a higher concentration of antibodies. Moving on to the *in vivo* section, the respective cells were encapsulated within different PSF MTAMs. In theory, due to the relatively small size of each PSF MTAM, which was about 0.5 cm × 1.5 cm, each mouse can be implanted with up to four PSF MTAMs, with different cells. In this study, we only utilized three different cells, namely, hybridoma as the source of the CEACAM6 antibody, PC 3 as a non CEACAM6 susceptible cancer cell line and MDA-MB-468 as a CEACAM6 antibody susceptible cancer cell line (Figure 4A). Overall, the 21 d *in vivo* test echoed the outcomes that were observed in the *in vitro* tests. The PSF MTAMs encapsulated hybridoma cells proliferated well throughout the entire 21 d in an increasing trend (Figure 4B). Conversely, the MDA-MB-468 cancer cell lines that were CEACAM6 antibody susceptible cancer cell line reduced in cell viability over time (Figure 4C–E). PC 3, a cancer cell line that was not susceptible to the CEACAM6 antibody, was revealed to show minimal differences in terms of cell viability when compared to that of the control, reinforcing the notion that the PSF MTAMs under *in vivo* conditions were excellent substrates that did not hamper the diffusion of molecules across the lumen wall, ultimately affecting the overall proliferation and viability. 

**Figure 5:**
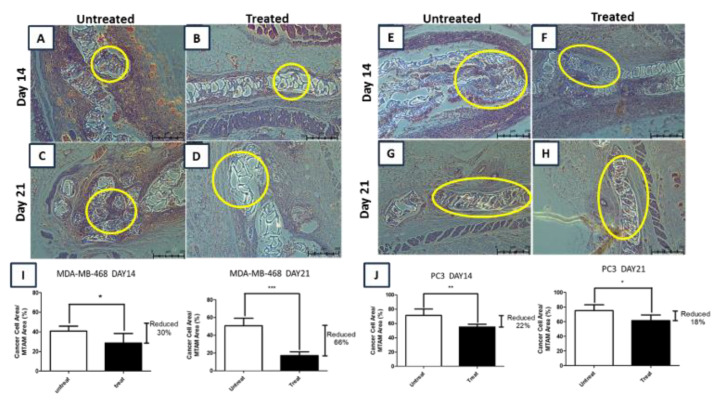
Day 14 and 21 of H&E staining images of MDA-MB-468 (**A**–**D**) and PC 3 (**E**–**H**); and the corresponding viabilities (**I** and **J**). When compared to control, a reduction in total cell area reduction of 30% (MDA-MB-468) and 21% (PC 3) were registered on day 14; and by day 21, the reduction increased to 68% (MDA-MB-468), while the reduction reduced to 18% (PC 3). In the case of MDA-MB-468 that was treated with hybridoma cell culture supernatant extract, almost no cells were observable by day 21 (**D**). In contrast, a large quantity of cells was still observable in PC 3 (**H**).

H&E staining of the recovered PSF MTAMs encapsulated cells again reinforced the findings of the *in vitro* and *in vivo* outcomes. In the untreated groups of the PC 3 and MDA-MD-468, cancer cell lines (Figure 5A,C,E,G) clearly indicated that the lumens of the PSF MTAMs were filled with these respective cells, and the viability of these respective cells increased slightly/remained the same compared to the duration of the experiment prolonged. This suggested that the PSF MTAMs not only had sufficient space for cell growth, but the unique microstructures were extremely beneficial in terms of supporting cancer cell line proliferation. Comparing the total cell area in H&E stains of MDA-MB-468 (Figure 5I) and PC 3 (Figure 5J), the reduction of cancer cell line viability was most significant of the breast cancer cell line, while PC 3 registered minimal differences. This reinforces the notion that the hybridoma cells that were encapsulated within the PSF MTAMs and encapsulated together as seen in Figure 4A did not only proliferated well, but were also functional in the release of sufficiently highly quantity of CEACAM6 antibodies in a continuous fashion. Additionally, the PSF MTAMs did not hinder any diffusion of the antibodies into the lumens of the respective PSF MTAMs. Lastly, the surrounding regions around the implanted PSF MTAMs did not reveal any signs of inflammations, confirming the biocompatibility nature of the material was not altered despite being dissolved in organic solvents and ultimately electrospun.

## 4. Discussion

The majority of the benefits of the PSF MTAMs acting as a cell culture substrate were derived from the micron/sub-micron scale microstructures. However, as the PSF MTAMs ultimately forms an arrayed of one-to-one connected fibers, which resulted in a membrane the size of 0.5 cm × 1.5 cm (Figure 1), which allowed it to be easily manipulated and used by technical personnel. The cell loading process into the respective lumens of the PSF MTAMs was conducted by utilizing the capillary action, which easily siphoned a droplet of cell suspension (10 µL). Furthermore, the PSF MTAMs were mechanically sound and capable of being worked on easily. To further enhance the value and ease of use of our PSF MTAMs, different biocompatible dyes can be easily added to the polymer solution prior to the electrospinning process. This resulted in differently colored PSF MTAMs that can be utilized as identification/differentiation between treatment groups in a study.

From the perspective of being a cell culture substrate that is capable of eliciting functional response of hybridoma cells, where increased antibody concentration were registered, the data demonstrated that the PSF MTAMs were superior to that of the standard TCPs (Figure 2). Despite registering a higher degree of cell viability when the hybridoma cells were culture in TCPs, the hybridoma cells that were encapsulated within the PSF MTAMs were not too far off, and more importantly, were able to continuously invoke a greater secretion of CEACAM6 antibody (Figure 2B). Primarily, this was because of the unique micron scale microstructures that mimicked a 3D cell culture system and the material (PSF) used in the fabrication of MTAMs, which in turn influenced the biocompatibility, cell signaling and gene and protein expression [24,31,32,33]. In addition to the 3D cell culture surface provided by the PSF MTAMs, the presence of homogenously distributed pores along the entire surface resulted in a ‘semi-rough’ surface, which functioned and mimicked nanostructures/nanotopographies, and ultimately enhanced cellular proliferation [34]. This in turn mediated cell attachment and proliferation, which functioned much like the basal membrane of cells that were responsible for the regulation of cellular function and support [24,35]. Another potential explanation for such observations was through the encapsulation of hybridoma cells within the lumens of PSF MTAMs, a protective effect of the membrane wall that reduced the susceptibility of the hybridoma cells to chemical, pH, temperature and medium composition changes [36,37].

In terms of diffusion of nutrients and waste across the lumen wall, the micron scale lumen wall (Figure 1D), which registered a value of 3.0 ± 1.0 μm, did not hamper this critical function. This was demonstrated in the *in vitro* and *in vivo* experiments, where the cells encapsulated within the PSF MTAMs proliferated well with the diffusion of CEACAM6 antibody which was about 150 kDA, which was about 0.1 µm when converted [38,39]**.** Unhindered throughout these experiments, as well as demonstrated in our previous work, the lumen walls of the PSF MTAM allowed excellent diffusion of both small and large molecular weight molecules across the lumen wall [34]. 

The dysregulation via overexpression of the immunoglobulin Carcinoembryonic antigen-related cell adhesion molecule 6 (CEACAM6), which is expressed on epithelial and myeloid surfaces, is oncogenic in nature; it modulates abnormal cell growth, cell differentiation, cell death and the resistance of anti-cancer agents [40,41]. As such, the data in Figure 3 and Figure 4 echoed each other and demonstrated that in the presence of the antibody (anti-CEACAM6) produced by the hybridoma cells in both the *in vitro* and *in vivo* setting, the cancer cell lines with CEACAM6 receptors such as A549 and MDA-MB-468 registered a reduction in overall cellular viability as opposed to the cancer cell line PC 3, which does not possess any antigen sites [30]. This outcome correlated with the outcome of work by several groups, which revealed that anti-CEACAM6 antibodies would inhibit the migration, invasive nature of tumor cells and adhesion through the interference of homo/hetero-typic binding which in turn invalidated the anoikis resistance (anchorage independent growth) of lung adenocarcinoma [40,42,43], while inhibiting the cellular proliferation, migration and invasive nature of triple negative breast cancer via the SRC and AKT signaling pathways [40,44]. In the presence of CEACAM6 antibody derived from the hybridoma cell culture extract, the MDA-MB-468 cancer cell line registered a greater reduction in cell viability when compared to the A 549 cancer cell line (Figure 3). Primarily, this observation was a result of a higher expression of CEACAM6 that was presence on the surfaces of MDA-MB-468; this in turn made it highly susceptible to the CEACAM6 antibody [30,44,45].

In the *in vivo* experiments (Figure 4 and Figure 5), normal mice were utilized instead of nude mice, as the PSF MTAMs provided a protection barrier against the host immune system. Uniquely, the PSF MTAMs enabled the implantation of multiple cancer cell lines, as each cancer cell line was isolated within their respective PSF MTAMs without affecting the diffusion of molecules across the ultra-thin lumen wall (Figure 1) [24,27,34]. As outlined earlier, the PSF MTAMs as cell culture substrates performed very well where the hybridoma cells and the untreated groups revealed excellent cell viabilities (Figure 4). This observation was further reinforced in the H&E staining of the recovered PSF MTAMs, which indicated that the lumens of the PSF MTAMs were fully occupied in terms of cell area (Figure 5). In addition to cell viability, the ability of the PSF MTAMs to invoke the secretion of Ceacam6 antibodies were also observed in Figure 4. As the *in vivo* experiment progressed into the 21st day, CEACAM6 antibody susceptible cells (MDA-MB-468) revealed a continuous reduction of cell viability (Figure 4C–E). This suggested that the hybridoma cells that were encapsulated within a separate PSF MTAM, located in the vicinity also continuously secreted CEACAM6 antibodies throughout this study. Furthermore, the diffusion of these secreted CEACAM6 antibodies were not hampered in any way by the presence of the PSF MTAMs. The outcome of the *in vivo* study echoed that of the *in vitro* study; similar findings were found in the H&E stained images of the recovered PSF MTAMs.

Unlike traditional xenograft models, which were normally limited to having one cell sample implanted in a single animal, encapsulated hybridoma cells, PC 3 and MDA-MB-468 cancer cell lines were encapsulated in their respective PSF MTAMs and subcutaneously implanted into the same Balb/C mouse; this was made possible by the ability to isolate the respective cells as well as protecting these cells from the attacks of the host’s immune system. Since immunotherapy generally involves a myriad of complex interactions [46], the ability to study the interactions between different cells is of utmost importance in the research and development of cancer immunotherapies. Additionally, the amount of animal utilized in models can be significantly reduced, and this resonates well with the recent emphasis of 3Rs in the use of animals in studies [47].

Future work related to this study would be to conduct prolonged *in vivo* study to determine the survivability and the ability to sustain the release of CEACAM6 antibody by hybridoma cells encapsulated within PSF MTAMs for at least 6 months to 12 months. The therapeutic effects of these antibodies, development of immunity against these antibodies by cancer cells and the potential side effects should also be included. 

## 5. Conclusions

This study demonstrated that PSF MTAMs were superior as a 3D cell culture substrate capable of eliciting higher production of CEACAM6 antibody. Additionally, this system showcased its ability to protect the cells encapsulated within from stress sources and host’s immune attacks without compromising the diffusion of nutrients, waste and/or metabolic products, namely CEACAM6 antibodies, which were critical in this study. The hybridoma cells successfully survived under *in vivo* conditions and continuously released sufficiently high levels of CEACAM6 antibodies; moreover, they were capable of suppressing CEACAM6 expressing tumor cells viability. In total, the evidence derived from this study suggested that the PSF MTAMs system is capable of being applied as an implantable and more importantly removable (if side effects were observed) encapsulated cell therapy for the fight against cancer.

## Supplementary Materials

The following are available online at https://www.mdpi.com/2077-0375/10/5/80/s1. Figure S1: SEM of the cross section of the heat sealed electrospun PSF MTAM which revealed no openings, forming a continuous seal on the micron/sub-micron scale; Figure S2: *In vitro* culture of MDA-MB-468 (top) and PC 3 (bottom) in TCPs and PSF MTAMs. Both culture substrates revealed an increasing viability across time; Figure S3: Immunohistochemistry staining of the respective cancer cells; MDA-MB-468 (top) and PC 3 (bottom). Clearly, PC-3 revealed no immunofluorescence signal when targeted fluorescent antibodies targeting CEACAM 6 were used.
