# Supplementary material for: Microtube Array Membrane (MTAM)-Based Encapsulated Cell Therapy for Cancer Treatment
Source: Membranes (Basel). 2020 Apr 26;10(5):80. doi: 10.3390/membranes10050080 (PMC7281484; doi:10.3390/membranes10050080)
Supplement: Supplementary file 1 [file membranes-10-00080-s001.pdf]

Microtube Array Membrane (MTAM) Based Encapsulated Cell Therapy for Cancer Treatment

Chee Ho Chew [1], Chih-Wei Lee [2,†], Wan-Ting Huang [3], Li-Wei Cheng [4], Amanda Chen [5], Tsai-Mu Cheng [6], Yen-Liu Liu[7], and Chien-Chung Chen [6,8,9,10,11,*]

Supplementary Figures:

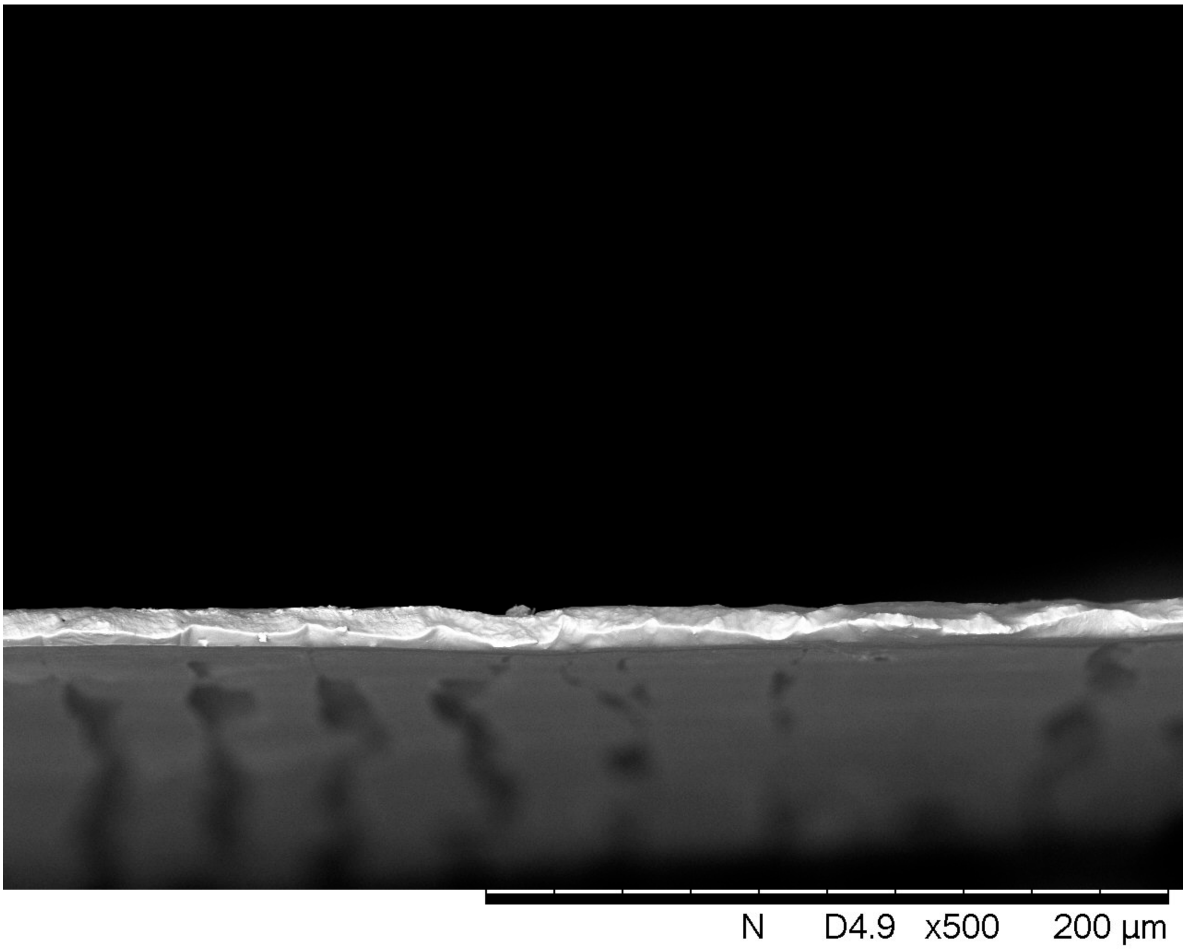

Supplementary figure 1: SEM of the cross section of the heat sealed electrospun PSF MTAM which revealed no openings, forming a continuous seal on the micron/sub-micron scale.

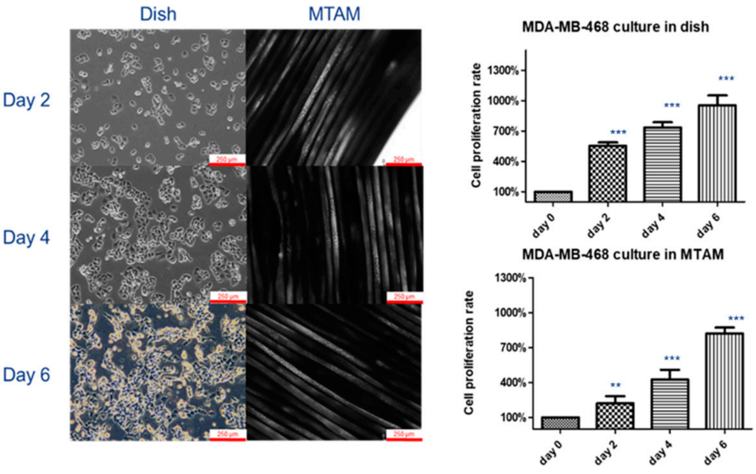

1.

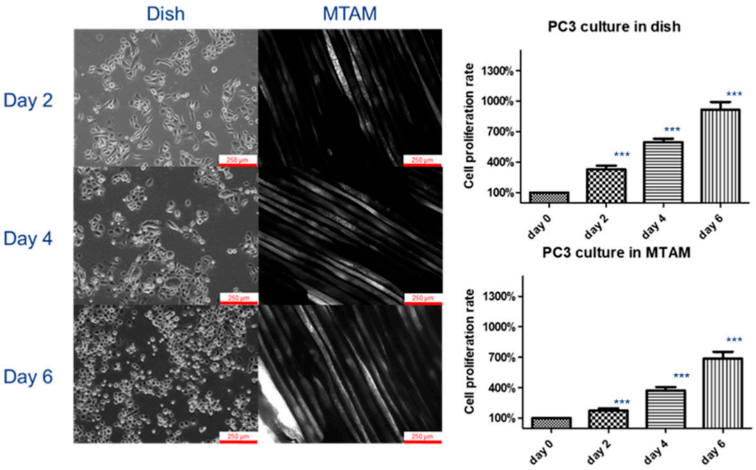

2.

Supplementary figure 2: *In vitro* culture of MDA-MB-468 (top) and PC 3 (bottom) in TCPs and PSF MTAMs. Both culture substrates revealed an increasing viability across time.

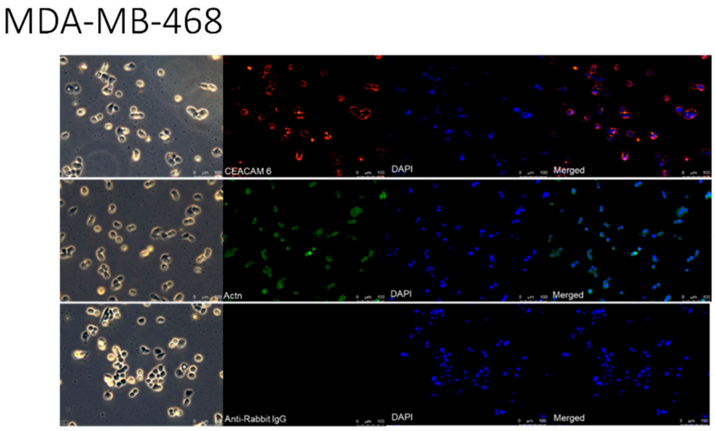

3.

PC3

4.

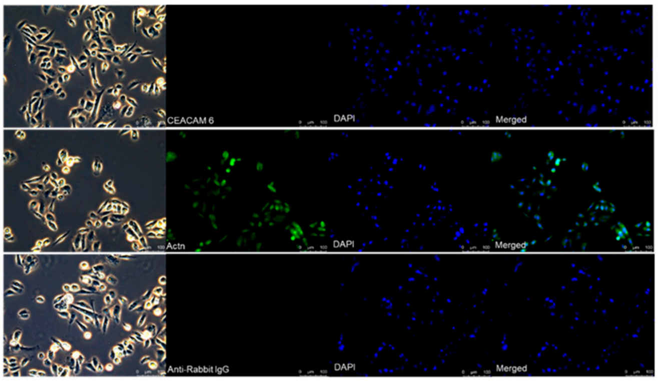

Supplementary figure 3: Immunohistochemistry staining of the respective cancer cells; MDA-MB-468 (top) and PC 3 (bottom). Clearly, PC-3 revealed no immunofluorescence signal when targeted fluorescent antibodies targeting CEACAM 6 were used.
